# Whole Genome Scan Uncovers Candidate Genes Related to Milk Production Traits in Barka Cattle

**DOI:** 10.3390/ijms25116142

**Published:** 2024-06-02

**Authors:** Wondossen Ayalew, Xiaoyun Wu, Getinet Mekuriaw Tarekegn, Tesfaye Sisay Tessema, Rakan Naboulsi, Renaud Van Damme, Erik Bongcam-Rudloff, Zewdu Edea, Min Chu, Solomon Enquahone, Chunnian Liang, Ping Yan

**Affiliations:** 1 Key Laboratory of Animal Genetics and Breeding on Tibetan Plateau, Ministry of Agriculture and Rural Affairs, Key Laboratory of Yak Breeding Engineering, Lanzhou Institute of Husbandry and Pharmaceutical Sciences, Chinese Academy of Agricultural Sciences, Lanzhou 730050, China; wondessenayalew9@gmail.com (W.A.); wuxiaoyun@caas.cn (X.W.); chumin@caas.cn (M.C.);; 2 Institute of Biotechnology, Addis Ababa University, Addis Ababa P.O. Box 1176, Ethiopia; 3 Scotland’s Rural College (SRUC), Easter Bush Campus, Roslin Institute Building, University of Edinburgh, Edinburgh EH25 9RG, UK; 4 Childhood Cancer Research Unit, Department of Women’s and Children’s Health, Karolinska Institute, Tomtebodavägen 18A, 17177 Stockholm, Sweden; 5 Department of Animal Biosciences, Bioinformatics Section, Swedish University of Agricultural Sciences, 75007 Uppsala, Swedenerik.bongcam@slu.se (E.B.-R.); 6 Ethiopian Bio and Emerging Technology Institute, Addis Ababa P.O. Box 5954, Ethiopia; zededeaget@gmail.com

**Keywords:** Barka cattle, milk production traits, selection signature, whole genome sequencing

## Abstract

In this study, our primary aim was to explore the genomic landscape of Barka cattle, a breed recognized for high milk production in a semi-arid environment, by focusing on genes with known roles in milk production traits. We employed genome-wide analysis and three selective sweep detection methods (*ZF_ST_*, *θπ* ratio, and *ZHp*) to identify candidate genes associated with milk production and composition traits. Notably, *ACAA1*, *P4HTM*, and *SLC4A4* were consistently identified by all methods. Functional annotation highlighted their roles in crucial biological processes such as fatty acid metabolism, mammary gland development, and milk protein synthesis. These findings contribute to understanding the genetic basis of milk production in Barka cattle, presenting opportunities for enhancing dairy cattle production in tropical climates. Further validation through genome-wide association studies and transcriptomic analyses is essential to fully exploit these candidate genes for selective breeding and genetic improvement in tropical dairy cattle.

## 1. Introduction

Cattle, one of the most economically significant livestock species globally, continue to play a vital role in agriculture and various cultural practices. Their domestication has driven a complex interplay of natural and artificial selection, shaping their adaptive traits and breed specialization [1]. Influenced by diverse ecosystems and climates, natural selection drove cattle to acquire unique adaptive characteristics. Concurrently, artificial selection refined these breeds for enhanced productivity and specific functions [2,3]. These dual processes have created diverse cattle breeds, finely specialized for distinct regions, exhibiting various adaptations, production capacities, and other phenotypic traits.

The African continent is widely acknowledged as a primary reservoir of cattle diversity [4,5]. Unlike commercially selected Western breeds, natural selection has predominantly influenced African cattle genomes [4,6]. Consequently, despite their adaptive traits, indigenous breeds’ production and reproduction performances remain poorly characterized [7]. Although African cattle breeds have often been regarded as less productive when compared to the intensively selected commercial breeds, some African breeds have the potential for improvement in dairy and beef traits, alongside their notable adaptability [8]. For example, recent genomic studies have pinpointed genes associated with milk production, composition traits, and meat attributes in various African indigenous cattle breeds [6,9,10]. These insights lay the basis for further in-depth research into the genomic characteristics of African cattle breeds for production traits.

Understanding the genetic potential of locally adapted dairy cattle breeding and improvement is crucial for sustainable milk production. This is particularly significant as temperate dairy breeds are more vulnerable to changing climates and environments, making their introduction to low-input systems less sustainable. Milk production, a complex polygenic trait, is influenced by multiple genes [11] and environmental factors. Identifying candidate genes and genomic regions that modulate milk production traits is essential for enhancing milk-related characteristics [12]. To date, various genomic regions and candidate genes linked to milk production and composition traits have been identified through different approaches, including the candidate gene approach [13,14,15], whole genome sequencing [6,16,17], validation through GWAS [18,19,20], as well as expression profiling [21,22,23].

Among Ethiopia’s 28 recognized cattle populations [24,25], the Barka cattle, alternatively called Begait, is native to the hot and semi-arid part of northwestern Ethiopia and Southern Eritrea [8]. Besides its unique adaptive attributes, this cattle population is characterized by a well-developed udder, long teats, and relatively high milk production performance [26,27]. Under an improved management system, it has been reported to achieve a daily milk yield of 12 L [28]. While the Barka cattle breed exhibits promising potential for milk production traits, genomic studies on this cattle breed have primarily emphasized genomic diversity and adaptive significance [29,30,31], with limited focus on its production potential. Thus, understanding the genetic determinants underlying milk production traits in Barka cattle is immensely important for sustainable dairy production, particularly in regions with typical hot climates and limited feed availability. We conducted a comparative analysis of Barka cattle as our focal test population with other breeds identified as low milk production yielders, including Ankole (2.2 L per day [32]) and the N’Dama breeds (1.5 L per day [33]). This study aims to identify the candidate genomic regions related to milk production and composition traits of Barka cattle in Ethiopia.

## 2. Results

### 2.1. Sequencing and Alignment Statistics

The individual genomes of 70 Ethiopian indigenous cattle breeds, namely Abigar (ABI), Barka (BAR), Boran (BOR), Fellata (FEL), Fogera (FOG), and Horro (HOR), were sequenced to an average coverage of approximately 14×. These genomes were then subjected to joint genotyping alongside publicly available genomic data from African Sanga (Ankole), African taurine (N’Dama), and a commercial cattle breed (Holstein) for comparative analysis (Tables S1 and S2). Burrows-Wheeler Aligner with Maximal Exact Matches algorithm (BWA MEM) [34] was employed for read alignment against the taurine reference genome sequence ARS-UCD1.2. The alignment process achieved an average alignment rate of 97.04%, covering 98.49% of the reference genome (Table S2).

### 2.2. Population Structure and Relationships

**Figure 1:**
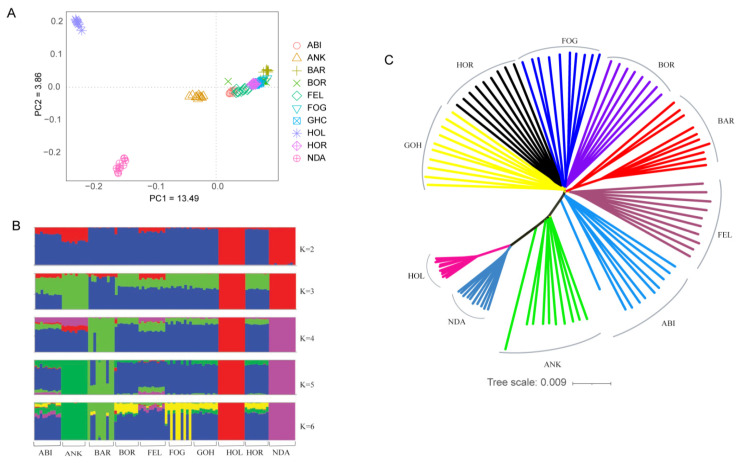
Population structure and relationships of Ethiopian cattle. (**A**) PCA plot. (**B**) Admixture plot showing breed proportions at K = 2–6. (**C**) Neighbor-joining tree constructed based on genetic distance.

Principal component (PC) and admixture analysis were employed to investigate the genetic structure of Ethiopian cattle breeds compared to reference populations, including Holstein, N’Dama, and Ankole cattle. PC1 and PC2 explained 13.49% and 3.86% of the total variation, respectively (Figure 1A). Regardless of their geographic location, the Ethiopian cattle populations demonstrated a notable genetic relationship, forming a distinct cluster within the analysis. On the other hand, Holstein and N’Dama cattle breeds exhibited separate and well-defined genetic clusters, with the Ankole breed positioned between the taurine (Holstein and N’Dama) and the Ethiopian cattle populations. This suggests possible genetic admixture or shared ancestry between Ankole cattle and the taurine and zebu populations. In line with the PCA plot, the admixture analysis revealed that Ethiopian cattle populations exhibited distinct differences from N’Dama and Holstein cattle breeds when the number of ancestral populations (K) was set to 2. However, at the lowest cross-validation error (K = 4) (Figure S1), Barka cattle showed some level of unique patterns of admixtures (Figure 1B). Furthermore, the unrooted NJ tree also corroborated the results obtained from the PC and admixture analysis (Figure 1C).

### 2.3. Genetic Diversity and Linkage Disequilibrium Decay

**Figure 2:**
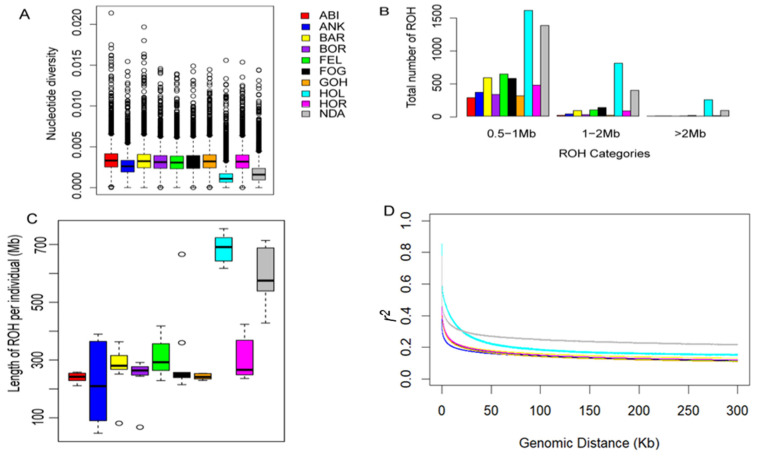
Summary of genomic variation statistics. (**A**) Genome-wide nucleotide diversity distribution of each breed in 100 kb windows with 50 kb increments. (**B**) The distributions of ROH categories in each breed. (**C**) The length of ROH in each breed. (**D**) Genome-wide LD decay of each breed.

Our analysis revealed that the levels of nucleotide diversity within the Ethiopian cattle populations were comparable to one another yet notably higher when compared to the reference populations (Figure 2A). This finding underscores the substantial genetic diversity present in Ethiopian cattle in contrast to the reference breeds. To evaluate the ROH patterns of Ethiopian cattle, the length of ROH was classified into three size classes: 0.5–1 Mb, 1–2 Mb, and >2 Mb. Ethiopian zebus primarily exhibited ROHs ranging from 0.5 to 1 Mb, while taurine breeds displayed higher ROH levels across all size categories (Figure 2B). Similarly, the length of ROH was higher in taurine breeds than in Ethiopian zebus (Figure 2C). In all cattle breeds, the highest r^2^ values were observed at short distances (<10 kb) with a gradual decrease as the physical distance between SNPs increased (up to 200 kb). Beyond the 200 kb threshold, a stable pattern of r^2^ values was consistently observed (Figure 2D). These observations align with the ROH results, where taurine breeds exhibited the highest r^2^ values, potentially influenced by a combination of artificial selection and reduction in effective population size. Conversely, the lower ROH length and r^2^ values in Ethiopian cattle support the greater genomic diversity, as indicated by the higher nucleotide diversity (Figure 2A). Furthermore, despite their phenotypic and geographic variations, population differentiation within Ethiopian cattle populations was consistently lower than the differentiation observed among non-Ethiopian cattle populations (Table 1).

### 2.4. Signatures of Selection in Barka Cattle

**Figure 3:**
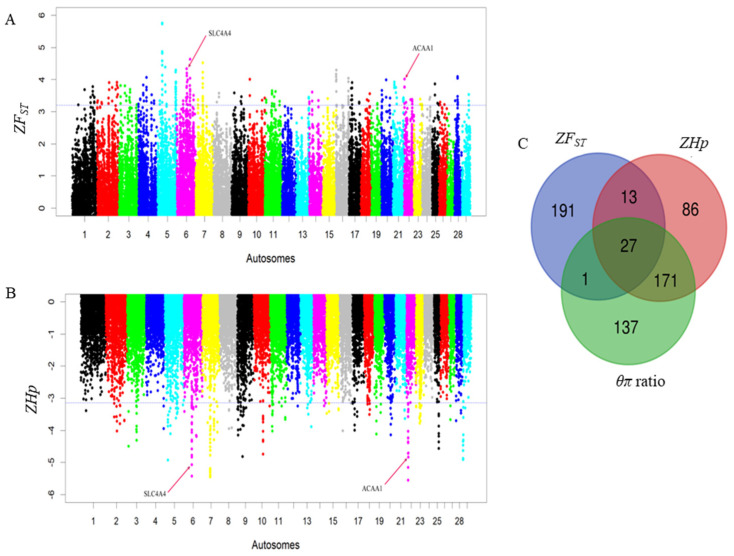
Genomic landscape depicting signatures of positive selection in the Barka cattle breed. (**A**) Pooled heterozygosity (*Hp*) at the threshold of *ZHp* = −3.14. (**B**) Population differentiation (*F_ST_*) at the threshold of *ZF_ST_* = 3.2. (**C**) Venn diagram showing the genes overlapping among Z*F_ST_*, *θπ* ratio, and Z*Hp* selection scan methods.

**Figure 4:**
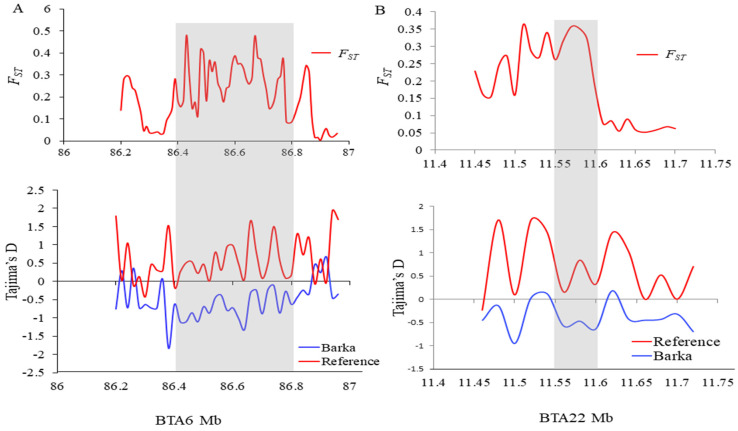
Population differentiation (*F_ST_*) and Tajima’s D plots. (**A**) *ACAA1* and (**B**) *SLC4A4* genes genomic regions.

To elucidate selective sweeps likely associated with milk traits in Barka cattle, we compared Barka with reference breeds abroad. In this particular study, Barka cattle were considered the test population. In contrast, Ankole and N’Dama cattle breeds were used as reference populations to detect selection signatures associated with milk production traits despite their differences in adaptation and other characteristics. A total of 232, 297, and 336 protein-coding genes were detected in Barka cattle using *ZF_ST_* (Table S3), *ZHp* (Table S4), and *θπ* ratio (Table S5) analyses, respectively. Across all selection scan analyses, 27 protein-coding genes were found to be shared (Figure 3C), among which three genes (*ACAA1*, *P4HTM*, and *SLC4A4*) were potentially associated with milk production and composition traits (Table 2, Figure 3A,B). Evidence for negative Tajima’s D scores and high *F_ST_* signals of *SLC4A4* (Figure 4A) and *ACAA1* genes (Figure 4B) suggested a strong positive selection of Barka cattle in these genomic regions.

### 2.5. Functional Annotations of Putative Selection Sweeps

To elucidate the functional relevance of genes identified as putative selection signatures in Barka cattle, we combined the Ensembl ID of candidate genes detected by all three selection scan methods and performed functional enrichment analyses on the online DAVID tools using *Bos taurus* as background. Candidate genes exhibiting highly analogous functions (*p* ≤ 0.05 and a fold enrichment ≥ 1.2) were classified into significant GO terms (Table S6). Based on the literature survey, positive regulation of phosphorus metabolic process (GO: 0010562), nucleocytoplasmic transport (GO: 0006913), monoatomic anion transport (GO: 0006820), and positive regulation of cell population proliferation (GO: 0008284) were the most significant GO terms (Table 3).

## 3. Discussion

### 3.1. Genetic Diversity, Relationships, and Population Structure

Characterizing genetic diversity and population structure is essential to reveal cattle populations’ adaptive and productive potential. These insights have profound implications for guiding future genetic improvement and conservation efforts [52]. The principal component (PC) and admixture analyses differentiate Ethiopian cattle from Ankole and African and European taurine breeds (Figure 1A,B). However, the Barka cattle breed exhibits unique genetic patterns distinct from other Ethiopian cattle breeds. This cattle breed is believed to have originated from the initial zebu introgression into Africa and exhibits distinctive genetic patterns reflecting their early adaptation in the region [25,30,53].

In contrast, the newly formed Zenga breeds (Fogera and Horro) and other zebus introduced during the second wave of zebu introgression following the rinderpest epidemic exhibit a different genetic signature [25,53]. This divergence in genetic patterns between Barka and Zenga cattle reflects their separate historical contexts and introgression events, supporting the hypothesis that the timing and circumstances of zebu cattle introductions have left lasting imprints on the genetic makeup of these breeds. Unlike the reference breeds, Ethiopian cattle exhibited notably higher levels of nucleotide diversity, affirming the higher genomic diversity within these cattle breeds. Our analysis also identified a limited occurrence of extended runs of homozygosity (ROH) (Figure 2B,C) and a slower rate of linkage disequilibrium (LD) decay in Ethiopian cattle compared to the reference breeds (Figure 2D). These low levels of ROH and LD decay align with prior research on other zebu cattle, indicating low selection pressure within these cattle breeds [6,31,54].

### 3.2. Candidate Genes Associated with Milk Production and Composition Traits

Milk production and composition traits are fundamental determinants of dairy cattle profitability, exerting a substantial influence on the economic viability of dairy enterprises. Although many African cattle breeds are not intensively selected for dairy characteristics, some indigenous breeds have displayed favorable dairy traits [6]. Among these breeds, the Barka cattle breed, found in the semi-arid lowlands of northwestern Ethiopia, is commonly recognized for its relatively higher milk-producing potential [8,28]. In contrast, the Ankole and N’Dama cattle breeds are characterized as poor milk producers [32,33]. Therefore, conducting a comparative genome analysis with the Barka breed as the test population and the Ankole and N’Dama breeds as reference populations is an effective strategy for identifying genomic regions that govern milk production and composition traits.

#### 3.2.1. Milk Production Traits

Milk production traits, encompassing milk yield, composition, and other relevant parameters, are complex and polygenic traits influenced by many genetic factors. In recent years, advancements in genomics and bioinformatics have opened new horizons for uncovering the genetic basis of milk traits. This study identified interesting genes modulating milk production traits in Barka cattle (Table 1). *ATP1B2*, encoding a subunit of the sodium–potassium pump critical for ion transport across cell membranes, has shown a positive correlation with milk yield and heat resistance [47]. Notably, the genetic variations within the second and fourth introns of *ATP1B2* have been observed as significant determinants of 305-day milk yield, milk fat content, and milk protein content in Chinese Holstein cows [14]. These findings underscore the pleiotropic effect of ATP1B2 on both tropical adaptation (heat resistance) and various milk traits. It is interesting to note that *GMDS* (GDP-Mannose 4,6-Dehydratase), located on BTA23 (51.11–51.16 Mb), could potentially have a functional role as a QTL for milk yield due to its involvement in fructose biosynthesis [42]. Fructose, as a critical component of various metabolic processes, may play a pivotal role in the energy balance of dairy cattle, which is closely tied to milk yield [55].

The *SLC4A4* gene on BTA6 exhibited a strong positive selection signal (*F_ST_* = 4.5) in Barka cattle (Table 2 and Figure 3A). This gene plays a pivotal role in regulating active glucose transport. It has previously been recognized as a candidate gene associated with milk yield traits [20]. Its significance is especially evident in milk synthesis, where the glucose uptake by mammary epithelial cells represents a crucial step with direct implications for milk production [21]. The positive selection signals observed in this gene are further corroborated by notably lower Tajima’s D (Figure 4A). Gene functional enrichment analysis revealed that *SLC4A4* is associated with various GO terms (Table 3 and Table S6). Notably, it is prominently linked to the positive regulation of the phosphorus metabolic process (GO:0010562), which is primarily responsible for milk production traits [6]. Another noteworthy candidate gene is *HNRNPL*, which is associated with alternative splicing and mRNA transport and has demonstrated a positive correlation with milk yield [38]. This gene activates *eNOS* splicing and influences *NOS3*, which modulates nipple erection, suggesting its potential significance as a critical marker for milk yield traits [38,56].

#### 3.2.2. Milk Fat Content

The milk composition holds significant importance within the dairy industry due to its direct influence on the nutritional profile and economic value of milk and various dairy products. Milk fat content is one of the compositional qualities, and its synthesis is a complex process regulated by a network of genes. Our analysis identified genes related to milk fat content, such as *ACACA*, *FABP3*, and *PRKG1*, in Barka cattle (Table 1). The *ACACA* gene, which encodes the Acetyl-CoA Carboxylase enzyme, plays a role in fatty acid metabolism. In dairy cattle, it’s associated with milk fat synthesis [22,57]. When the *ACACA* gene is more active, it can lead to an increased conversion of acetyl-CoA to malonyl-CoA, resulting in an increased synthesis of fatty acids. This can ultimately lead to higher milk fat content in dairy cattle [35]. The FABP3 gene, situated on BTA2, is a member of the fatty acid binding protein (*FABP*) family. It exhibits predominant expression in the mammary glands of cattle and has been implicated in regulating milk fat synthesis [18,58]. The upregulation of the FABP3 gene is pivotal in stimulating dairy cattle’s milk fat synthesis signaling pathway [36].

Additionally, the polymorphisms of the *FABP3* gene have been found to influence milk fat and protein content in Jersey cattle [59]. The *PRKG1* gene on BTA26 was identified as a candidate in our analysis. It plays a crucial role in adipocytes, facilitating triacylglycerol hydrolysis, releasing fatty acids and glycerol, and contributing to lipolysis [43]. The GWAS and transcriptional profiling studies support the assumption that the *PRKG1* gene regulates milk fatty acid metabolism in dairy cattle [18,43].

#### 3.2.3. Milk Protein Content

Understanding the genetic factors controlling milk protein synthesis is crucial for a comprehensive understanding of milk composition in cattle. Our analysis has revealed several candidate genes, including *ANGPT1*, *CRIM1*, *P4HTM*, and *PLEC*, which offer intriguing avenues for investigating their roles in milk protein content, particularly in Barka cattle. *ANGPT1*, also known as Ang1, has been previously recognized as a ligand for the TEK Receptor Tyrosine Kinase (TEK) and is associated with the *PI3K-Akt* signaling pathway, a pathway known to correlate with milk protein synthesis [37]. *ANGPT1*’s involvement in vascular network development, as demonstrated in mouse studies [60], suggests a potential role in facilitating nutrient transport and supply to the mammary gland, which is essential for milk synthesis. Identifying *ANGPT1* as a candidate gene within our study has significant implications for understanding the genetic factors influencing milk protein production in Barka cattle.

Cysteine-rich transmembrane BMP regulator 1 (*CRIM1*) is another candidate gene encoding a protein characterized by cysteine-rich repeat structures, along with IGF-binding protein motifs and insulin-like growth factor binding protein motifs [61]. The presence of these motifs implies its potential involvement in insulin-related pathways known to influence milk protein gene expression, casein synthesis, and nutrient uptake in mammary glands [50,51]. Furthermore, genome-wide analysis in Holstein cattle identified the presence of the *PLEC* gene within selection sweeps, suggesting its association with critical candidate genes involved in milk protein expression [20,23]. The *P4HTM* gene has been linked to milk protein in dairy cattle [41] and sheep [62]. These findings imply the potential roles of these candidate genes in influencing milk protein content in Barka cattle and provide a basis for further investigations into their specific mechanisms and contributions to milk composition. As milk production and composition traits are complex polygenic traits, the detection of false positive and false negative results in selection signature analysis is expected. Therefore, validating these findings through alternative methods, including GWAS, the candidate gene approach, and gene expression analysis, is imperative.

#### 3.2.4. Mammary Gland Development

Mammary gland development is a pivotal determinant determining milk production in dairy cattle [63]. Our selection signature analysis identified four candidate genes, *ACAA1*, *CSF1*, *ERBB3*, and *MED1*, which strongly correlate with mammary gland development and function (Table 2). The strong associations of these genes with mammary gland development were further confirmed by functional enrichment analysis linked to several important GO terms (Table 3 and Table S6). *ACAA1* is a crucial enzyme involved in fatty acid metabolism, regulating breakdown and synthesis processes while influencing pathways related to fat and casein synthesis in mammary epithelial cells [64]. Markedly, Deng et al. [40] revealed that *ACAA1* overexpression leads to enhanced mammary epithelial cell proliferation and increased secretion of triglycerides and β-casein, underlining its significant regulatory role in mammary gland activity, particularly in the synthesis of essential milk components.

The colony-stimulating factor 1 (*CSF1*) is another promising gene that significantly regulates macrophage migration and functions in various tissues, including the mammary gland [44]. The influence of this gene on macrophage activity within mammary tissue is noteworthy, as macrophages play a crucial role in tissue remodeling, immune defense, and milk synthesis during lactation [65]. *ErbB3*, also known as *HER3*, is a receptor tyrosine kinase and a member of the epidermal growth factor receptor (*EGFR*) family. It plays a significant role in mammary gland development and function, particularly during pregnancy and lactation [45,46]. *ErbB3* is activated by neuregulin (*NRG1*), a growth factor secreted by mammary epithelial cells, and it plays a critical role in stimulating the proliferation and differentiation of mammary epithelial cells [66]. This process is essential for forming alveoli, the milk-producing structures within the mammary gland. The *MED1* gene, a mediator complex subunit, is located on BTA19 (39.84–39.86 Mb). A previous study has reported that the *MED1* gene is indispensable in mammary gland development and lactation [67,68]. Its involvement, particularly in conjunction with estrogen receptors (ERs), is primarily observed during the developmental phase of the mammary gland in puberty and in facilitating luminal cell differentiation [48].

## 4. Materials and Methods

### 4.1. Study Populations and Sequencing

**Figure 5:**
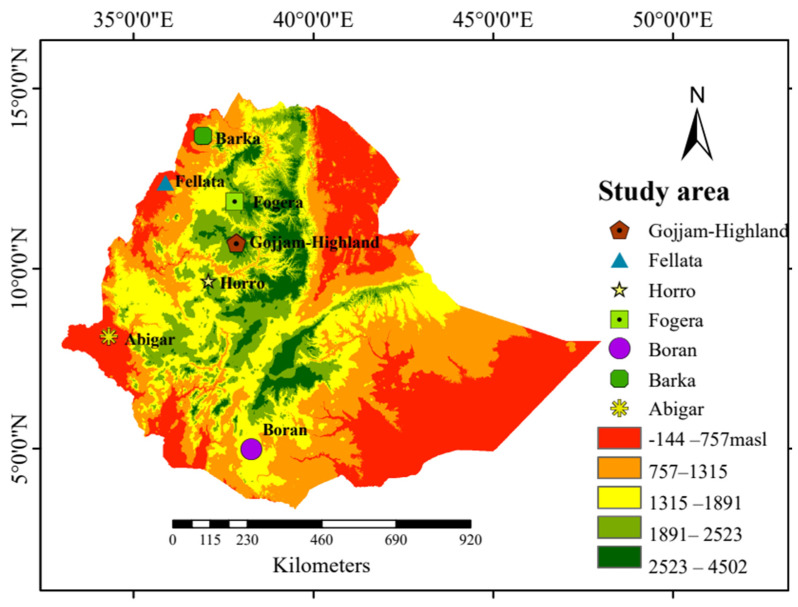
Geographical map of Ethiopia indicating the sampling locations of the cattle populations with the altitude. Gojjam = Gojjam-Highland.

Seventy blood samples were collected from seven Ethiopian cattle populations, with 10 unrelated cattle from each population. These cattle were from diverse agroecological regions within their natural breeding habitats (Figure 5). Genomic DNA was extracted from 5 μg of blood using a Tiangen genomic DNA extraction kit based on the manufacturer’s protocols (TIANGEN Biotech, Beijing, China) and DNA libraries were prepared by ligating paired-end adapters and performing 150 bp PCR amplification. Subsequently, the amplicons were sequenced using the MGI-SEQ 2000 platform, generating a length of 150 bp paired-end reads. For comparison purposes of the selection sweeps and genetic diversity, we used 30 publicly available reference samples (Ankole, Holstein, and N’Dama; 10 samples from each breed) [69,70] obtained from public databases (Table S1). Among the seven cattle breeds sequenced, 20 samples, comprising 10 from the Abigar and 10 from the Barka cattle breeds, were previously included in our publication by Ayalew et al. [71].

### 4.2. Alignment and Variant Identification

The paired-end reads of the seven Ethiopian cattle breeds and the retrieved reference sequences underwent adapter trimming using Trimomatic v0.39 [72]. The filtered reads were aligned against the cattle reference genome ARS-UCD1.2 [73] using default settings with BWA-MEM 0.7.17-r1188 [34]. After alignment, the output in SAM format was converted to BAM format, indexed, and sorted by coordinates using Samtools version 1.6 [74]. The resultant BAM files were processed to mark duplicates using Picard Tools 2.27.4 (https://broadinstitute.github.io/picard/, accessed on 8 March 2022). Subsequently, the non-duplicated individual BAM files underwent base quality recalibration. They were further processed through the ‘HaplotypeCaller,’ ‘CombineGVCFs,’ and ‘GenotypeGVCFs’ functions of GATK version 4.3.0.0 for calling raw SNPs [75], ultimately generating a jointly genotyped VCF file. The Variant Quality Score Recalibration (VQSR) process within the same software was employed on the raw variants to refine the set of variants. Validated SNPs from the 1000 Bull Genome Project were used for this calibration. The ‘SelectVariant’ procedure was applied to retrain variants meeting a 99% truth sensitivity threshold and remove low-quality variants. Finally, 36,527,967 autosomal SNPs were used for downstream analysis.

### 4.3. Genetic Diversity and Linkage Disequilibrium 

The average nucleotide diversity (*π*) and population genetic differentiation (*F_ST_*) were assessed using high-quality autosomal SNPs. These SNPs were examined within non-overlapping 100 kb windows with a step size of 50 kb across the entire set of bovine autosomes using VCFtools version 0.1.15 [76]. To assess the genome-wide linkage disequilibrium (LD) within each breed, we computed the average r2 values for pairwise markers using the PopLDdecay software v.3.42 [77] with default settings. SNPs with a minor allele frequency (MAF) of greater than 0.05 were considered in this analysis. The number and size of homozygosity (ROH) runs were estimated for each breed using the methods described in a previous study [78].

### 4.4. Population Structure and Relationships

After filtering out low-quality sequence data, the high-quality autosomal SNPs underwent additional screening, applying a minor allele frequency (MAF) threshold of 0.05. SNPs with more than 10% missing genotypes were eliminated using VCFtools [76]. Subsequently, the remaining SNPs were subjected to pairwise linkage disequilibrium pruning using Plink 1.9 [79] with the parameters of --indep-pairwise 50 10 0.2. These processes resulted in 1,344,914 SNPs used for Principal component (PC), admixture, and phylogenetic analyses. PC analysis was performed using the Plink 1.9 software package [79]. The resulting eigenvectors were visualized through ggplot2 in R [80]. To estimate the levels of admixture within the study populations, we employed ADMIXTURE version 1.3.0 software [81], running the analysis for values of K from 1 to 10 and plotted by ggplot2. Furthermore, we constructed an unrooted Neighbor-Joining (NJ) tree based on pairwise genetic distances, and the tree was visualized using Interactive Tree Of Life (iTOL) v.6.8.1 [82].

### 4.5. Selective Sweep Analysis and Annotation

In cattle, domestication and artificial selection have reduced nucleotide diversity and changes in allele frequencies. To elucidate genomic regions under selection and explore the differences between promising dairy breeds, such as Barka, and poor milk-producing cattle breeds (Ankole and N’Dama), two complementary comparative selection sweep analysis approaches were employed. First, we estimated the population differentiation (*F_ST_*) [83] with a sliding window of 100 kb and 50 kb step size using VCFtools [76]. Then, the nucleotide diversity of the test population (Barka) and reference population (Ankole and N’Dama) was computed using VCFtools commands (--window-pi 100,000 --window-pi-step 50,000). The *θπ* ratio between the test population and reference populations was calculated as ln (*θπ*, Barka/*θπ*, Ankole, and N’Dama). In addition, using the same software, window, and step size, a within-population pooled heterozygosity (*ZHp*) selection scan was performed in Barka cattle. The genomic regions that show high *ZF_ST_* values (top 0.5% of *ZF_ST_* distribution), low levels of nucleotide diversity (top 0.5% for *θπ* ratio), and extremely low *ZHp* scores (the bottom 0.5% of *ZHp* distributions) were considered to represent genomic regions under selection. Tajima’s D and *F_ST_* statistics were computed for candidate genes using VCFtools [76].

### 4.6. Functional Analysis of the Candidate Genes

The candidate genomic regions identified by the three complementary approaches (*ZF_ST_*, *ZHp*, and *θπ* ratio) were annotated using the Ensembl Biomart annotation tool (http://useast.ensembl.org/index.html, accessed on 25 September 2023) [84], using the ARS-UCD1.2 cattle reference genome [36]. To better understand the molecular functions of the candidate genes, we performed enrichment analyses using the Gene Ontology (GO) and Kyoto Encyclopedia of Genes and Genomes (KEGG) databases through the online DAVID tools [85]. The *p*-values for gene enrichment were subjected to correction using the Benjamini–Hochberg method to control the false discovery rate (FDR). Significantly enriched GO and KEGG pathways were determined by considering only those GO and pathways where the corrected *p*-value fell below the threshold of 0.05.

## 5. Conclusions

With their rich genetic diversity and remarkable adaptability, indigenous cattle breeds hold significant potential for enhancing milk production and composition traits. Through this study, we have unveiled several candidate genes that shed light on the underlying mechanisms influencing milk production in Barka cattle. These findings offer new avenues for further exploration into the genetic determinants of milk traits in indigenous breeds. Identifying candidate genes associated with milk yield, fat content, protein content, and mammary gland development underscores the complex genetic architecture underlying dairy traits. *ATP1B2*, *SLC4A4*, *GMDS*, and *HNRNPL* are notable genes implicated in milk production, highlighting their potential role in enhancing Barka cattle’s dairy productivity. Understanding the genetic basis of milk traits is crucial for developing targeted breeding programs to improve dairy productivity and preserve indigenous cattle breeds. Given the challenges posed by climate change and the increasing demand for milk production, harnessing the resilience and productivity of native cattle breeds is essential for sustainable dairy production, particularly in regions with limited feed resources and hot climates. Further validation and in-depth functional investigations of these candidate genes are warranted to elucidate their precise roles in milk production and composition.

## Figures and Tables

**Table 1:** Population differentiation (*F_ST_*) between Ethiopian and reference cattle breeds.

Breeds	ABI	ANK	BAR	BOR	FEL	FOG	GOH	HOL	HOR	NDA
ABI										
ANK	0.055									
BAR	0.062	0.122								
BOR	0.026	0.0827	0.057							
FEL	0.0275	0.0795	0.061	0.0334						
FOG	0.0275	0.0891	0.065	0.0192	0.042					
GOH	0.0219	0.074	0.055	0.0115	0.029	0.019				
HOL	0.062	0.25	0.349	0.3513	0.316	0.349	0.332			
HOR	0.0211	0.0733	0.058	0.014	0.03	0.021	0.0061	0.336		
NDA	0.189	0.173	0.267	0.2594	0.222	0.259	0.2418	0.265	0.243	

ABI = Abigar, ANK = Ankole, BAR = Barka, BOR = Boran, GOH = Gojjam-highland, FEL = Fellata, FOG = Fogera, HOL = Holstein, HOR = Horro, NDA = N’Dama.

**Table 2:** Candidate genes detected using *ZF_ST_*, *θπ* ratio, and *ZHp* selection scan methods influencing milk production and composition traits in dairy cattle.

Methods	BTA	Start Position	End Position	Gene Name	Summary of Gene Function	References
*ZF_ST_*	2	122285620	122294666	*FABP3*	Milk fat	[35,36]
	2	1679994	1864849	*ARHGEF4*	Milk yield	[19]
	6	86381836	86809131	*SLC4A4*	Milk production	[20]
	14	56984054	57285247	*ANGPT1*	Milk composition traits	[37]
	18	48557658	48569498	*HNRNPL*	Milk yield	[38]
	21	65626343	65634828	*DLK1*	Milk protein and milk fat	[39]
	22	11581703	11607351	*ACAA1*	Mammary epithelial cell proliferation	[40]
	22	50983772	50997485	*P4HTM*	Milk traits	[41]
	23	51168216	51604000	*GMDS*	Milk production	[42]
	26	6899619	8313722	*PRKG1*	Milk fatty acid traits	[18,43]
*θπ* ratio	3	33488957	33509479	*CSF1*	Mammary gland development	[44]
	5	57215784	57236737	*ERBB3*	Mammary development	[45,46]
	6	86381836	86809131	*SLC4A4*	Milk production	[20]
	19	13441162	13726679	*ACACA*	Milk fat	[6,35]
	19	27364091	27369121	*ATP1B2*	Milk yield and milk composition	[14,47]
	19	39843840	39867840	*MED1*	Mammary gland development	[48]
	22	50983772	50997485	*P4HTM*	Milk traits	[41]
	22	11581703	11607351	*ACAA1*	Mammary epithelial cell proliferation	[40]
*ZHp*	6	86381836	86809131	*SLC4A4*	Milk production traits	[20]
	20	39873127	40265889	*ADAMTS12*	Milk production	[49]
	11	18812764	19022665	*CRIM1*	Milk protein	[50,51]
	14	56984054	57285247	*ANGPT1*	Milk composition traits	[37]
	15	56246885	56403904	*ACER3*	Mammary gland development	[41]
	22	50983772	50997485	*P4HTM*	Milk traits	[41]
	22	11581703	11607351	*ACAA1*	Mammary epithelial cell proliferation	[40]

**Table 3:** GO cluster annotation of candidate genes commonly detected by the Z*F_ST_*, *θπ* ratio, and *ZHp* selection scan methods.

Term	Count	*p*-Value	Fold Enrichment	Genes
GO: 0010562—positive regulation of phosphorus metabolic process	36	0.041	1.36	*CACUL1, DAB2IP, EPHA5, ETAA1, FXR2, FYN, MYD88, ROS1, SH3RF3, TYRO3, VRK3, ACVRL1, ANGPT1, BMPR2, CHI3L1, CSF1, DSTYK, ERBB3, FGF18, GDF9, HBEGF, HMGA2, HIPK2, INHBC, INHBE, IL23A, KIF14, LEPR, NCF1, RPS6KA5, SLAMF1, SLC4A4, SPPL3, STOML2, TP53, VCP*
GO: 0006913—nucleocytoplasmic transport	19	0.005	2.10	*ABCE1, POLDIP3, RANBP1, RANBP17, TRAF3IP2, YTHDC1, AHCYL1, BMPR2, FAM53C, HSPA9, KPNA6, MED1, NUTF2, NPM1, LOC511386, NUP133, NUP62, TCF7L2, TP53*
GO: 0001932—regulation of protein phosphorylation	46	0.033	1.35	*AKT1S1, CACUL1, DAB2IP, ETAA1, FKBP1A, FXR2, FYN, GPS2, MYD88, ROS1, SH3RF3, TIMP3, VRK3, ACVRL1, ANGPT1, BMPR2, CHI3L1, CSF1, CCNG1, CDK12, DSTYK, FGF18, QARS1, GDF9, HBEGF, HMGA2, HIPK2, INHBC, INHBE, IL23A, KIF14, NCF1, LEPR, NPM1, NUP62, PARD6A, PLEC, RPS6KA5, RNF41, STAT2, SLAMF1, SIRT2, SLIT2, SMPD3, TADA2A, TP53*
GO: 0006820—monoatomic anion transport	19	0.032	1.27	*ABCB11, ATP8A1, ATP8B3, ATP9A,* *ROS1, FABP3, GABRB1, LOC516849, SFRP4, SLC12A4, SLC22A11, SLC22A12, SLC23A1, SLC25A48, SLC38A3, SLC4A4, SLC4A8, SLC4A9, SLC7A6*
GO: 0008284—positive regulation of cell proliferation	32	0.017	1.54	*HTR1B, CACUL1, GNAI2, GLI1, LHX1, MYD88, POU3F3, SOX15, ACVRL1, ACER3, BMPR2, CSF1, EGR1, ERBB3, FGF18, GDF9, HBEGF, HMGA2, HIPK2, IL23A, KIF14, LDLRAP1, MZB1, MED1, NR4A1, NPM1, OTP, SLAMF1, SMPD3, TNC, TCF7L2*

## Data Availability

The raw sequencing data for the Ethiopian cattle samples are available from the Sequence Read Archive (SRA) with the Bioproject accession numbers PRJNA1053488 and PRJNA1059514. The accessions for the previously published datasets can be found in Table S1.

## Supplementary Materials

The following supporting information can be downloaded at: https://www.mdpi.com/article/10.3390/ijms25116142/s1.
