# Supplementary figures and images for: Whole Genome Scan Uncovers Candidate Genes Related to Milk Production Traits in Barka Cattle
Source: Int J Mol Sci. 2024 Jun 2;25(11):6142. doi: 10.3390/ijms25116142 (PMC11172929; doi:10.3390/ijms25116142)

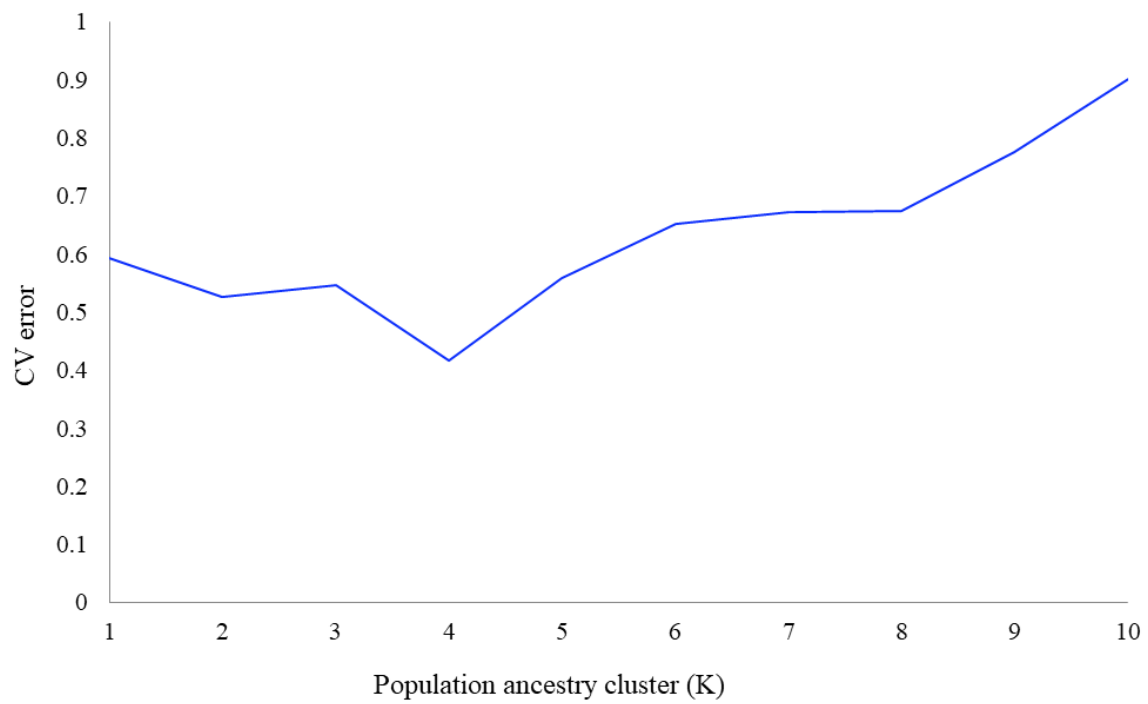

Figure S1 Distribution of the coefficients of error variance

Supplement: Supplementary file 1 [file ijms-25-06142-s001.zip › Supplementary Figure S1.pdf]
